# Platypnea-Orthodeoxia Syndrome: Presentation of a 90-Year-Old

**DOI:** 10.7759/cureus.68673

**Published:** 2024-09-04

**Authors:** Daniel Song, Michael Rydberg, Emily Cetrone

**Affiliations:** 1 Internal Medicine, University of North Carolina at Chapel Hill, Chapel Hill, USA

**Keywords:** transesophageal echocardiogram (tee), hypoxemia, quality of life, multidisciplinary discussion, platypnea orthodeoxia syndrome, goals of care, geriatric assessment, patent foramen ovale, orthodeoxia, platypnea

## Abstract

Platypnea-orthodeoxia syndrome (POS) is a clinical syndrome of dyspnea and hypoxemia that is exacerbated by sitting upright or standing and resolved with lying flat. Here, we discuss an initial presentation of POS as a result of a previously undiagnosed patent foramen ovale (PFO) in a 90-year-old man. Diagnosis of the PFO was limited by technically difficult transthoracic echocardiograms with inconclusive agitated saline studies. Transesophageal echocardiogram (TEE) with Doppler and agitated saline study was eventually diagnostic, and his symptoms resolved after percutaneous PFO closure. The diagnosis and treatment in this patient were complicated by his age, moderate dementia, and limited decision-making capacity. Although our patient was dependent for virtually all instrumental activities of daily living (iADLs), he and his family reported an excellent quality of life prior to presentation, and we anticipated he would be able to regain this post-procedurally, allowing us to advocate for TEE and subsequent PFO repair. In the geriatric population, special consideration must be taken to discuss the risks and benefits of extensive workup and treatment depending on the effectiveness and invasiveness of both; approaching these cases with this holistic approach can thus help guide their clinical course appropriately.

## Introduction

Platypnea-orthodeoxia syndrome (POS) is defined as dyspnea (platypnea) and hypoxemia (orthodeoxia) that worsens when sitting upright and improves with recumbency [1]. The diagnostic cutoff for the arterial desaturation is commonly defined as a greater than 4 mmHg drop in partial pressure of oxygen (PaO2) or greater than 5% drop in oxygen saturation (SpO2) when going from supine to upright [1-3]. As the name suggests, POS is a clinical syndrome, and should prompt providers to investigate underlying causes [2] which are broadly divided into intra-cardiac or extra-cardiac categories [1-3]. The terms were first coined in the 1960s-1970s [3], however the syndrome remains likely under-diagnosed. One 2012 review article only examined 188 patients across 105 articles [4]. Another 2022 review article included 231 articles but primarily case reports, small patient series, and reviews [3]. Here, we discuss a case of a 90-year-old man with no prior cardiac or pulmonary history, who presented with acute hypoxemic respiratory failure, found to have POS secondary to a newly diagnosed patent foramen ovale (PFO).

## Case presentation

A 90-year-old man presented to the emergency department after a mechanical fall at home and was found to be in acute hypoxemic respiratory failure. He had shortness of breath that progressed over several months, which the patient attributed to deconditioning due to little exercise at his assisted living facility. He denied cough or orthopnea, and had no known prior cardiac history. On exam, his SpO2 was 84% on room air. His lungs were clear to auscultation, and he had no lower extremity edema. Computed tomography angiography (CTA) Chest with contrast on admission saw no findings of pulmonary emboli, consolidation, pulmonary edema, or other acute cardiopulmonary process. Initial trans-thoracic echocardiogram (TTE) also showed normal left ventricular (LV) ejection fraction, but had limited cardiac windows.

While lying supine, he remained relatively asymptomatic and was able to wean to lower oxygen rates and eventually to room air. However, even mild exertion or sitting upright resulted in a desaturation to the 80s, requiring immediate increase in oxygen requirement. An arterial blood gas obtained during one of these episodes showed a pH of 7.41, arterial partial pressure of carbon dioxide (pCO2) of 34.2, and an arterial pO2 of 63 while on 5L O2 via nasal cannula, confirming true hypoxemia. Given D-dimer elevation to 1,661 on admission, a repeat CTA chest was obtained acutely which again showed no pulmonary emboli; however, this study noted suspicion for a PFO.

**Figure 1:**
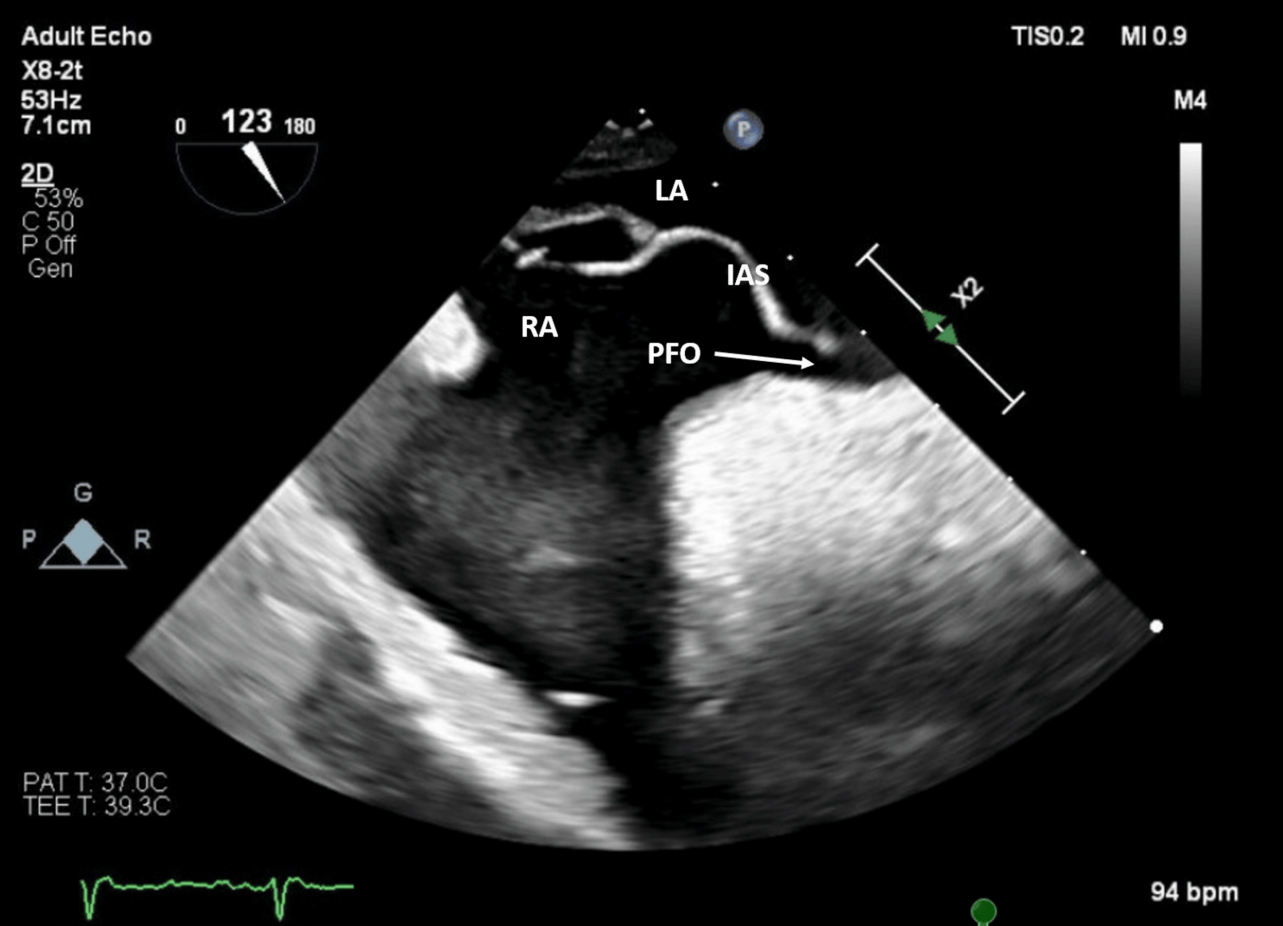
Trans-esophageal echocardiogram (TEE) demonstrating a patent foramen ovale with an aneurysmal interatrial septum RA = Right Atrium; LA = Left Atrium; IAS = Interatrial Septum; PFO = Patent Foramen Ovale

**Figure 2:**
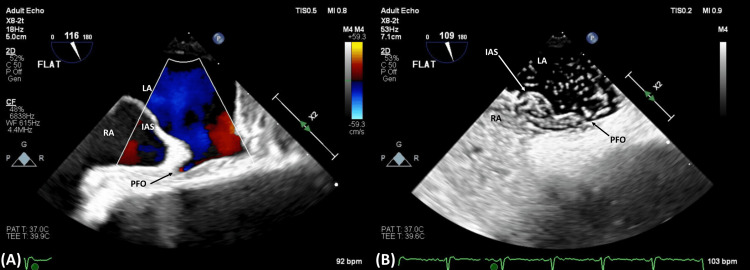
Trans-esophageal echocardiogram (TEE) while laying flat, demonstrating mild right-to-left shunting via PFO – (A) Doppler study; (B) agitated saline/bubble study RA = Right Atrium; LA = Left Atrium; IAS = Interatrial Septum; PFO = Patent Foramen Ovale

**Figure 3:**
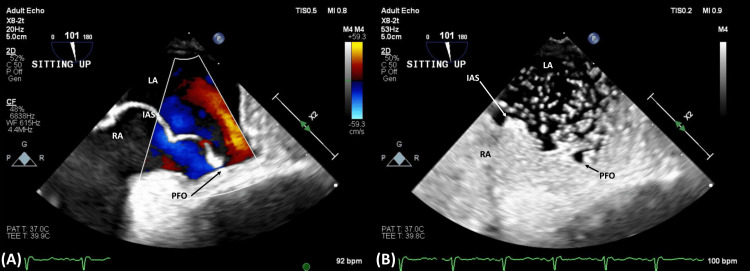
Trans-esophageal echocardiogram (TEE) while sitting upright, demonstrating more pronounced right-to-left shunting via PFO as compared to lying flat (Figure 2) – (A) Doppler study; (B) agitated saline/bubble study RA = Right Atrium; LA = Left Atrium; IAS = Interatrial Septum; PFO = Patent Foramen Ovale

Subsequent TTEs were obtained, but they were again limited due to technical difficulty, and were unsuccessful in visualizing any PFO on agitated saline studies. Given the high clinical suspicion for shunt physiology despite multiple negative TTEs, a multi-disciplinary discussion was held between geriatrics, pulmonology, and cardiology, regarding the utility of further diagnostic imaging. A trans-esophageal echocardiogram (TEE) was then obtained, which did reveal a PFO and an aneurysmal atrial septum (Figure 1), as well as intermittent moderate right-to-left shunting on color flow and grossly atrial-level positive bubble study: shunting was present while lying flat and more exaggerated with sitting up (Figures 2, 3).

The patient was taken to the catheterization lab for planned percutaneous PFO closure. A large tunnel PFO with adjacent fenestration through the septum primum was noted, with significant right-to-left shunting, as well as an aneurysmal septum primum. A 30mm Cardioform Septal Occluder (Gore, Flagstaff, AZ, USA) was implanted successfully, with subsequent negative TTE bubble study. The patient was weaned to room air without further respiratory distress, and he was eventually discharged to acute inpatient rehab. He established care in the Cardiology clinic after completing inpatient rehab, again with normal LV function, no valvular abnormalities, and no inter-atrial flow communication or intra-pulmonary shunt on repeat TTE, two to three weeks post-PFO closure.

## Discussion

Hypoxemia, the reduction in partial pressure of oxygen in the blood, has a broad differential diagnosis, that is commonly separated into V/Q mismatch, right-to-left shunts, diffusion impairment, hypoventilation, and low inspired oxygen partial pressure. For a majority of these etiologies, the addition of supplemental oxygen improves the hypoxemia. The exception is in right-to-left shunting, as there is complete bypass of ventilation, resulting in persistent hypoxemia despite supplemental oxygen [5]. In our patient, his hypoxemia initially improved without acute intervention and he was able to be weaned to room air while supine. However, as demonstrated on arterial blood gas (ABG), his hypoxemia was present despite oxygen therapy, and only resolved with recumbency. Thus, the positional nature of his symptoms was consistent with POS, and these additional clinical findings paired with the CT imaging were further suggestive of a right-to-left shunt via a PFO.

Presence of POS should prompt further diagnostic workup to determine the underlying etiology, as there is typically some structural alteration causing shunt physiology. Per a 2017 review article, the most common etiology is an intra-cardiac communication (n = 208/239, 97%); this most frequently will be a PFO, followed by an atrial septal defect (ASD) or atrial septum aneurysm (ASA) [1]. Other causes are broadly categorized as “extra-cardiac” shunts, commonly from intra-pulmonary arteriovenous (AV) malformations (9.2%) or ventilation perfusion mismatch from lung parenchymal disease (3.7%) [1]. Initial workup should focus on ruling out intracardiac shunt with echocardiography (TTE vs TEE) [1-3], with consideration of cardiac MRI if high enough clinical suspicion [1]. In our case, multiple TTE attempts were unsuccessful in diagnosing right-to-left shunts due to study limitations, but TEE was eventually diagnostic. Shunting that is noted in less than three beats via agitated saline/bubble study is typically suggestive of an intra-cardiac source, while extra-cardiac causes are noted within three-to-six beats [1-3]. Perfusion lung scans (V/Q scans, CTA Chest) can be diagnostic of pulmonary AV malformations. If imaging is negative, providers can consider hepato-pulmonary syndrome versus parenchymal lung disease as a diagnosis of exclusion [3]. Treatment depends on underlying etiology, such as repair of cardiac defect versus embolization of pulmonary AV malformations [1,3].

As with all other therapies, risk and benefits must be discussed with patients and tailored to their specific clinical scenario. Especially in the advanced geriatric population, patients’ goals of care should be explored, including degree to which they desire procedures and diagnostic testing, baseline functional status, and anticipated quality of life with or without the procedure in question [6]. In this case, it was important to assess our patient with a comprehensive geriatric understanding. Our 90-year-old patient had moderate dementia and was dependent for all of his independent activities of daily living (iADLs), starting to require assistance even with dressing. In addition, he did not have decision-making capacity, requiring us to primarily rely on discussions with his family. However, upon our evaluation, he had a very high quality of life prior to presentation, actively spending time with his family, and was a valued member of his community. The two end-points of the hospitalization were either to pursue diagnostic workup and possibly reverse his hypoxemia, versus discharging with hospice services due to his symptomatic dyspnea despite supplemental oxygen. Because of the potential regain of quality of life, we and the family felt it was appropriate to pursue TEE imaging and defect repair if indicated, as the benefits well outweighed the risks. However, if the TEE were non-diagnostic, all parties were in agreement that pulmonary testing and further imaging may not be worthwhile, since any findings would require much more aggressive procedures. As discussed above, our patient had a successful repair with good anticipated quality of life moving forward. The greatest strength in our approach was the multi-disciplinary nature of our discussions, involving not only Cardiology, Pulmonology, and Geriatrics in determining the most appropriate diagnostic/therapeutic interventions, but also the family in assisting with goals of care and substituted decision-making. We acknowledge that such sub-specialist expertise and the resources to proceed with testing and procedures in a timely manner may not always be available. In addition, family members may also not always be available for, nor amenable to, such in-depth, medically complex conversations, especially when patients are clinically decompensating rapidly. However, our comprehensive approach towards geriatric evaluation and discussion regarding goals of care is both accessible and applicable to all medical providers. 

## Conclusions

We discuss here a case of POS that presented as hypoxemia in a 90-year-old man due to a PFO, which was percutaneously repaired, with complete resolution of hypoxemia. POS should be considered in positional-dependent hypoxemia and can be due to a variety of etiologies. The initial workup should begin with TTE/TEE to evaluate for intra-cardiac causes, followed by perfusion imaging if non-diagnostic to evaluate for pulmonary malformations or other extra-cardiac etiologies. In the geriatric population, decisions regarding testing and treatments can be complicated by age, dementia, and limited decision-making capacity. Keeping all of these complex factors in mind can guide the clinical course appropriately: this often will require discussions with their family, friends, or healthcare decision-makers who can speak to their goals of care, functional status, and quality of life prior to hospitalization. We present our patient as a successful example of such an approach, as we were more aggressive in diagnosing and treating his hypoxemia, despite his age/dementia, because he had such high potential quality of life to be regained from doing so.
